# Mosquito-Independent Transmission of West Nile virus in Farmed Saltwater Crocodiles (*Crocodylus porosus*)

**DOI:** 10.3390/v12020198

**Published:** 2020-02-11

**Authors:** Gervais Habarugira, Jasmin Moran, Agathe M.G. Colmant, Steven S. Davis, Caitlin A. O’Brien, Sonja Hall-Mendelin, Jamie McMahon, Glen Hewitson, Neelima Nair, Jean Barcelon, Willy W. Suen, Lorna Melville, Jody Hobson-Peters, Roy A. Hall, Sally R. Isberg, Helle Bielefeldt-Ohmann

**Affiliations:** 1 School of Veterinary Science, University of Queensland, Gatton, Qld 4343, Australia; g.habarugira@uq.net.au; 2 Centre for Crocodile Research, Noonamah, NT 0837, Australia; research@crocresearch.com.au; 3 School of Chemistry and Molecular Biosciences, University of Queensland, St Lucia, Qld 4072, Australiacaitlin.obrien@uqconnect.edu.au (C.A.O.); willy.suen@csiro.au (W.W.S.); j.peters2@uq.edu.au (J.H.-P.); 4 Australian Infectious Diseases Centre, University of Queensland, St Lucia, Qld 4072, Australia; 5 Berrimah Veterinary Laboratories, NT 0828, Australia; steven.davis@menzies.edu.au (S.S.D.); Lorna.Melville@nt.gov.au (L.M.); 6 Queensland Health, Forensic and Scientific Services, Public Health Virology, Coopers Plains, Qld 4108, Australia; Sonja.Hall-Mendelin@health.qld.gov.au (S.H.-M.); Jamie.McMahon@health.qld.gov.au (J.M.); Glen.Hewitson@health.qld.gov.au (G.H.); Neelima.Nair@health.qld.gov.au (N.N.); Jean.Barcelon@health.qld.gov.au (J.B.)

**Keywords:** West Nile virus, saltwater crocodile, water-borne transmission

## Abstract

West Nile virus, Kunjin strain (WNV_KUN_) is endemic in Northern Australia, but rarely causes clinical disease in humans and horses. Recently, WNV_KUN_ genomic material was detected in cutaneous lesions of farmed saltwater crocodiles (*Crocodylus porosus*), but live virus could not be isolated, begging the question of the pathogenesis of these lesions. Crocodile hatchlings were experimentally infected with either 10^5^ (*n* = 10) or 10^4^ (*n* = 11) TCID_50_-doses of WNV_KUN_ and each group co-housed with six uninfected hatchlings in a mosquito-free facility. Seven hatchlings were mock-infected and housed separately. Each crocodile was rotationally examined and blood-sampled every third day over a 3-week period. Eleven animals, including three crocodiles developing typical skin lesions, were culled and sampled 21 days post-infection (dpi). The remaining hatchlings were blood-sampled fortnightly until experimental endpoint 87 dpi. All hatchlings remained free of overt clinical disease, apart from skin lesions, throughout the experiment. Viremia was detected by qRT-PCR in infected animals during 2–17 dpi and in-contact animals 11–21 dpi, indicating horizontal mosquito-independent transmission. Detection of viral genome in tank-water as well as oral and cloacal swabs, collected on multiple days, suggests that shedding into pen-water and subsequent mucosal infection is the most likely route. All inoculated animals and some in-contact animals developed virus-neutralizing antibodies detectable from 17 dpi. Virus-neutralizing antibody titers continued to increase in exposed animals until the experimental endpoint, suggestive of persisting viral antigen. However, no viral antigen was detected by immunohistochemistry in any tissue sample, including from skin and intestine. While this study confirmed that infection of saltwater crocodiles with WNV_KUN_ was associated with the formation of skin lesions, we were unable to elucidate the pathogenesis of these lesions or the nidus of viral persistence. Our results nevertheless suggest that prevention of WNV_KUN_ infection and induction of skin lesions in farmed crocodiles may require management of both mosquito-borne and water-borne viral transmission in addition to vaccination strategies.

## 1. Introduction

West Nile virus (WNV) is a mosquito-transmitted flavivirus that produces a potentially fatal disease in humans, horses, birds and alligators and has been associated with outbreaks of viral encephalitis in Africa, Europe, and the Americas [1]. Although the Kunjin strain of WNV was initially considered a separate species in the flavivirus genus, subsequent studies revealed that it shared a high degree of antigenic and genetic homology to other WNV strains [2,3,4], prompting the International Committee for Taxonomy on Viruses (ICTV) to classify Kunjin virus as a subtype of WNV. Until 2011, the relatively benign WNV_KUN_ had only been associated with a few cases of non-fatal encephalitis in humans and a handful of equine cases, since it was first isolated in 1960 [5]. However, in 2011 an emerging strain of WNV_KUN_ (referred to as NSW2011) caused a major outbreak of equine encephalitis in SE Australia [4,6].

When WNV first entered the Americas in 1999 (the WNV_NY99_ strain), much effort went into identifying hosts and reservoirs for the virus, and reptiles became a focus of attention. Serological surveys detected antibodies to WNV in farmed Nile crocodiles (*Crocodylus niloticus*) in Israel, wild and farmed Morelet’s crocodiles (*C. moreletii*) in Mexico as well as wild and captive American alligators (*Alligator mississippiensis*) in Florida and free-ranging alligators in Louisiana (reviewed in [7,8]). WNV_NY99_ was found to be associated with neurological and gastrointestinal disease and high mortality in farmed alligators in Georgia, Louisiana and Florida. Alligators with clinical signs exhibited very high WNV loads in liver, spleen, intestine and brain—the same tissues displayed severe pathological changes [9,10]. Skin lesions were also noted in animals surviving the acute infection, generally appearing 4–5 weeks after the acute disease, but it was not until later that a direct association between the skin lesions, known as “pix” or “Lymphohistiocytic proliferative cutaneous lesions” (LPCL), and WNV-infection was made [10,11].

In late 2016, similar skin lesions (pix) were discovered in farmed saltwater crocodiles (*Crocodylus porosus*) in the Northern Territory of Australia, and WNV_KUN_ viral RNA was detected by qRT-PCR [12]. These lesions severely diminish the value of the skin, with some farms in the Northern Territory observing lesions in almost half the crocodiles harvested. Farming saltwater crocodiles in Northern Australia is an emerging primary industry, currently worth more than AU$100 million per year with quality crocodile skins highly sought by the international fashion industry. However, lost production due to WNV_KUN_ infection is estimated to cost the industry more than AU$10 million/year. Of note is that the appearance of the lesions was not preceded by any other apparent clinical signs in the animals, suggesting that the virus–host relationship between WNV_KUN_ and *C. porosus* is different to that observed between WNV_NY99_ and alligators. This prompted us to further characterize the virus strain detected in the lesions and the infection in hatchling saltwater crocodiles. 

The mode of transmission of WNV_KUN_ to crocodiles is likely to be via the bite of infected mosquitoes. This is consistent with preliminary vector prevalence studies conducted on or near crocodile farms in Northern Australia, that showed high numbers of *Culex annulirostris*, the major mosquito vector of WNV_KUN_ in Australia [13]. Another incriminated WNV_KUN_ vector, *Culex quinquefasciatus* [14], was also found breeding in some of the crocodile rearing ponds (unpublished findings of the Northern Territory Medical Entomology unit). Herons and egrets (*Ardeidae*)—present in large numbers on crocodile farms scavenging for crocodile food waste—are the main recognized vertebrate hosts of WNV_KUN_ [5] and are likely to be involved in the initiation and maintenance of the transmission cycle on crocodile farms. In addition, some crocodile farms contain breeding pens to produce a constant supply of eggs and hatchlings. These swamp-like environments provide ideal conditions for mosquito breeding and attract large populations of these water birds [15]—the perfect scenario for WNV_KUN_ transmission. However, an additional role for infected crocodiles in transmitting the virus to mosquitoes, i.e., as amplifying hosts, has not been investigated. Another potential transmission route is via water fecally contaminated with WNV_KUN_, particularly in pens containing a high density of animals. Indeed, this has been shown to occur for WNV on alligator farms in the USA [10]. However, while alligators experience a necrotizing enteritis with WNV-shedding in the fecal material during acute infections [10], there has been no evidence that saltwater crocodiles are similarly clinically affected. In this report, we describe the full genome sequence of the crocodile-derived virus and its genetic relationship to other WNV_KUN_ strains as well as the outcome of both direct experimental infection and indirect (contact) virus transmission in juvenile (hatchling) saltwater crocodiles. Our results suggest that despite absence of clinical signs or pathological lesions in the gastrointestinal tract, WNV_KUN_ is indeed shed into the water and can be transmitted directly to other animals in close contact. The implications are that in order to protect farmed crocodiles from developing WNV_KUN_-induced skin lesions, causing financial costs to a locally important industry, a two-pronged approach must be taken: control of the mosquito-bird-crocodile transmission cycle, and the crocodile-to-crocodile transmission, the latter probably best aided by vaccination. 

## 2. Materials and Methods

### 2.1. Cell Culture and Virus

African green monkey (Vero) and *Aedes albopictus* larvae (C6/36) cells were cultured as previously described [16]. The isolation, propagation and characterization of the equine pathogenic WNV_KUN_ outbreak strain (NSW2011 - isolate E667) has previously been described in detail [4,6]. An additional two passages in BSR (derivative of BHK-1 hamster kidney cells) and C6/36 cells, respectively, were performed at Berrimah Veterinary Laboratories (BVL) prior to use for inoculation. 

To assess replication of the NSW2011 strain of WNV_KUN_ in *C. porosus* derived cell lines, 3-CPK and 1-LV cells [17] were infected at a multiplicity of infection (MOI) of 0.1, alongside C6/36 and Vero cells. The crocodile derived cells were maintained in Medium 199 with 10–15% fetal bovine serum (FBS), 50 U·mL^−1^ penicillin, 50 µg·mL^−1^ streptomycin, and 2 mM l-glutamine. All inoculated cells were incubated for five days and the culture supernatants harvested and titrated on C6/36 mosquito cells. The viral titers (TCID_50_ infectious units/mL) were determined by fixed-cell ELISA and calculated as per Reed and Muench [18]. The prototype strain of WNV_KUN_ (MRM61C, passage unknown, C6/36-derived stock) was used for comparison in these experiments.

Assessment of WNV_KUN_ growth kinetics in 1-LV and a chicken fibroblast cell line (DF-1) was performed in 24-well plates (Costar, Corning). The wells were coated with poly-d-Lysine (PDL), 1 mg/mL (Sigma-Aldrich Pty. Ltd., North Ryde, NSW, Australia), by incubation at 37 °C for 1 h followed by aspiration of the PDL-solution and two rounds of washing with sterile cell culture grade water. The plates were air-dried for 1 hour and subsequently seeded with 10^5^ 1-LV or DF-1 cells per well. Following overnight incubation at 34 °C, the cells were infected with WNV_KUN_ at a MOI of 1. Following virus-adsorption for 2 h at room temperature with rocking, the supernatant was discarded and the cell monolayers washed three times with sterile PBS, after which each well received 1 mL of M199 medium supplemented with 5% FBS, PSG and 2.5 mM HEPES. The cells were incubated at 34 °C. For each time point, the supernatant from three infected wells and one mock-infected well were collected and stored at −80 °C until virus titration by TCID_50_-assay as described in Section 2.6.

### 2.2. Sequencing and Phylogenetic Analysis

RNA was extracted from crocodile lesions and screened for the presence of flaviviruses using the pan-flavivirus generic primers FU2/cFD3 binding to the conserved NS5 region [19]. One positive sample (D66) was sent for next generation Illumina sequencing on a HiSeq platform (Australian Genome Research Facility, Melbourne, Victoria) after initial identification of WNV by Sanger sequencing of the pan-flavivirus primers-derived amplicon.

The reads obtained were mapped to the published genomes of WNV_KUN_ and 222 reads corresponded to the crocodile-derived WNV. Selected regions of the viral genome remained unsequenced, so primers were designed (Table S1) to produce large amplicons from viral RNA template extracted by high-fidelity RT-PCR (SuperScript™ III One-Step RT-PCR System with Platinum™ Taq High Fidelity DNA Polymerase (Thermo Fisher Scientific Australia Pty Ltd, Scoresby, VIC, Australia)). These amplicons were sequenced by Sanger sequencing (Australian Genome Research Facility, Brisbane, Queensland). The whole genome of the crocodile-derived WNV was obtained following this method and was included in a nucleotide alignment with various WNV sequences, using MAFFT in Geneious v8.1.9. The alignment was then used to generate a maximum likelihood phylogenetic tree, using MrBayes 3.2.6 in Geneious v8.1.9 [20] with a Generalized Time Reversible substitution model, a rate variation with invariable proportion remaining gamma, 5 gamma categories and the sequence of Murray Valley Encephalitis virus (NC_000943) as an outgroup. The Markov Chain Monte Carlo settings were 1,100,00 chain length, 4 heated chains, 0.2 heated chain temp, 200 subsampling frequency, 100,000 burn in length and a random seed.

### 2.3. Experimental Animals and Housing 

All protocols were approved by the Charles Darwin University Animal Ethics Committee (Permit # A18004; 31 January, 2018 to 31 January, 2019). Thirty-nine hatchlings were obtained from four wild clutches incubated under standard conditions (32 ± 0.5 °C; 95%–100% humidity) at Darwin Crocodile Farm, Noonamah, Northern Territory, Australia [21,22,23]. On the day of hatch, each animal was scute cut for individual identification [15] and randomized between three pens. Each pen was 200 cm wide and 202 cm in length including a feed deck 30 cm wide tapering to a maximum water depth of 19.5 cm. These pens were housed in an enclosed building at BVL, to prevent natural infection by mosquitoes, and temperature controlled using thermostatically controlled air (32 °C ± 2 °C) and water (32 °C ± 1 °C) heaters (Hobo™ data Loggers Onset Computer Corporation, MA). The rear two-thirds of each pen was covered with black shade cloth to provide security and heat retention and a smaller hide-board was also provided for additional security. Crocodiles were fed to excess five times weekly with meat mince enhanced with 2% vitamin/mineral premix (Monsoon Crocodile Premix, Brisbane, Australia) and 1.5% calcium carbonate. Residual food was removed the following morning and pens were cleaned thoroughly with a chlorine-based detergent. Monthly water samples were taken to ensure no environmental WNV_KUN_ prior to infection.

### 2.4. Experimental Infection

Prior to infection, crocodiles were measured (head and total length [24]), blood sampled from the occipital sinus using a 23-gauge needle, and belly skins were photographed at one, two, three and four months post-hatching. At four months, the crocodiles were randomized into three treatment groups: 10^4^ infectious units (IU) (*n* = 11; infection controls = 6), 10^5^ IU (*n* = 10; infection controls = 6) and control (*n* = 7) as shown in Table 1. At that stage, with the exception of one animal in the control group, all crocodiles were seronegative for passively acquired (maternal) WNV_KUN_ specific antibodies. 

Crocodiles were infected with the different doses of WNV_KUN_ in a volume of 0.1 mL given by a 29-gauge insulin needle as a subcutaneous injection behind the hind leg. Post infection (p.i.) blood sampling (up to 200 µL; EDTA tubes), cloacal and oral swabs, and skin inspection were done daily, but on a rotating basis so that any one animal was only bled every three days for the first 21 days p.i. Subsequently, all animals were bled at scheduled termination (day 21 p.i.) and/or every two weeks until termination of the experiment. Whole blood and plasma samples collected were aliquoted in triplicate and immediately frozen at −80 °C for virus isolation and serology, including blocking ELISA and VNTs (see later).

The animals were clinically assessed on a daily basis, with special attention to activity, growth rate, and neurological signs. At day 21 p.i., 11 animals were terminated (three from the control group; four from the 10^5^ IU-group, three from the 10^4^ IU-group and one from the 10^4^ IU-in-contact group) and subjected to necropsy and tissue sampling (see below). The remaining 29 animals were terminated at three months p.i. (seven months of age) and also subjected to necropsy and tissue sampling.

### 2.5. RT-PCR, qRT-PCR and Sequencing

#### 2.5.1. Water

Monthly water samples were collected from each treatment pen prior to infection and daily post-infection. Water was collected in 60 mL syringes and pushed through a 0.45 µM nitrate cellulose paper using a Swinnex™ Filter Holder (Millipore, Merck, Bayswater, VIC, Australia). Viral RNA was extracted from the filters by the RNeasy PowerWater Kit following the manufacturer’s instruction (QIAGEN Pty Ltd, Chadstone Centre, VIC, Australia). 

#### 2.5.2. Cloacal and Oral Swabs

The throat and cloaca were swab sampled each time the animal was bled during the infection period. The cotton tips were stored in 350 µL phosphate buffered gelatin saline (PBGS). For the extraction, the cotton tip was lifted out of the PBGS and placed in a clean microcentrifuge tube. The extraction was conducted using the RNeasy Plus Mini Kit (QIAGEN) following the manufacturer’s instruction for tissue samples. 

All extractions were qRT-PCR processed using a previously described WNV_KUN_ detection protocol [19], using the forward primer AACCCCAGTGGAGAAGTGGA, reverse primer TCAGGCTGCCACACCAAA and probe 6FAM-CGATGTTCCATACTCTGG-MGB NFQ [25]. Using the Applied Biosystems’ MicroAmp Fast 96-Well Reaction Plate and 7500 Fasr Real-Time PCR system, water extracts were run against a WNV_KUN_ standard (Table S2), while swabs were assessed for presence/absence of viral RNA (cut-off at Ct ≥ 40 [19]).

#### 2.5.3. Skin Samples

Skin sections, 4 mm × 4 mm (or smaller) with suspect lesions, sampled at necropsy, were stored in sterile vials at −80 °C until processing. The samples were then placed into a beater tube (cryovial containing 0.3 g of 0.5 mm diameter Zirconia/silicon beads) with 900 µL of PBGS, beaten for 1 min 30 s on a Qiagen Tissue Lyser and centrifuged at 8000 rpm for 1 min. Using the manufacturer’s instructions for the MagMAX-96 Viral RNA Isolation Kit (Applied Biosystems, Thermo Fisher Scientific Australia Pty Ltd, Scoresby, VIC, Australia), 50 µL of the homogenized sample was processed for RNA purification. The isolated RNA was subsequently stored at −80 °C until subjected to the qRT-PCR as described above.

#### 2.5.4. Plasma Samples

Crocodile plasma was diluted in AVE (molecular grade water with preservative from QIAGEN) 1:4 and viral RNA was extracted on EZ1 Advanced XL instrument (Qiagen, Hilden, Germany) using the EZ1 Virus Mini Kit V 2.0 (Qiagen, Clifton Hill, Australia) according to the manufacturer’s instructions. For WNV_KUN_ RNA detection, Superscript III Platinum one-step quantitative qRT-PCR system (Invitrogen, Carlsbad, CA, USA) was used as per the manufacturer’s instructions and based on the methods described by Pyke et al. [19] with minor modifications (primer and probe concentrations). Primer and probe sequences were as described in the section on swab samples. Primers were used at a final concentration of 900 nM, probe at 150 nM. Detection of WNV_KUN_ specific RNA was performed in 20 µL reactions in a Rotor-Gene 600 real-time PCR cycler (Qiagen, Chadstone, VIC, Australia) with the following cycling conditions: one cycle at 50 °C for 5 min, one cycle at 95 °C for 2 min, and 50 cycles at 95 °C for 3 s and 60 °C for 30 s. Separate synthetic controls for WNV_KUN_ primers and probe and no template controls were included in each Rotor-Gene run as per [19]. A standard curve was generated to determine WNV_KUN_ IU equivalents from plasma qRT-PCR CT scores. Ten-fold dilutions of WNV_KUN_ (10^−1^ to 10^−7^) were simultaneously assessed for infectious titre by TCID_50_ assay (see Section 2.6) and levels of viral RNA by Taqman qRT-PCR. An exponential trend line was generated from the derived Ct scores and calculated IU of the standard dilution series using the Excel Growth Function. Infectious unit equivalents were then predicted for each plasma sample from their derived Ct scores (Figure S1).

### 2.6. Virus Isolation and Titration

Infectious virus titers in blood were determined by the TCID_50_ method as previously described [16]. Briefly, C6/36 cells were seeded in maintenance RPMI culture media supplemented with 5% FBS, 50 U·mL^−1^ penicillin, 50 µg·mL^−1^ streptomycin, and 2 mM l-glutamine into each well of a 96-well tissue culture plate (Costar, Corning) and incubated overnight at 28 °C, at which stage the cell monolayers were at 80% confluency. Plasma or whole blood samples were diluted in 10-fold serial dilutions in RPMI with 2% FBS, 50 U mL^−1^ penicillin, 50 µg·mL^−1^ streptomycin, and 2 mM l-glutamine. Fifty µL of the diluted samples were transferred in triplicate onto the subconfluent C6/36 cell monolayer and the plates were incubated at 28 °C. After five days, the cultures were terminated by discarding the supernatant or transferring it into a new 96-well plate and storing at 4 °C pending the fixed cell ELISA results. The cells were fixed overnight with 20% acetone supplemented with 0.02% bovine serum albumen (BSA) at 4 °C. The fixative was then discarded, and plates dried overnight at room temperature before fixed cell ELISA was performed using monoclonal antibody (mAb) 4G2 (specific for the flavivirus E protein) and or 4G4 (specific for epitope on the viral NS1 protein) as previously described [16].

### 2.7. Histopathology and Immunohistochemistry (IHC) 

Samples were harvested immediately after euthanasia from all major organs and tissues, including brain, eyes, lungs, heart, liver, kidneys, spleen, gastrointestinal tract (multiple segments), tongue, skeletal muscle and skin, and fixed in 10% neutral buffered formalin solution for 48 hours before being transferred into 70% ethanol for storage until trimming and routine processing for paraffin embedding. Bone-containing specimens were decalcified by incubation in 8% formic acid for five days prior to trimming and paraffin embedding. Four micrometer thick sections were stained with hematoxylin and eosin and examined on a Nikon Eclipse 51 E microscope. Digital microphotographs were taken using a Nikon DS-Fi1 camera with a DS-U2 unit and NIS elements F 4.60 software. Images are reproduced without manipulations other than cropping and adjustment of light intensity.

Serial sections (4 µm) were cut from formaldehyde-fixed, paraffin-embedded samples and subjected to IHC-labeling as previously described in detail [26]. Briefly, following deparaffinization, antigen retrieval (EDTA, pH 9) and several blocking steps, the sections were incubated with the flavivirus NS1-specific mouse mAb 4G4 or a mixture of 4G4 and the E-protein specific mAb 4G2 as primary antibody followed by visualization of the binding using the DAKO Envision kit, specific for mouse immunoglobulin (Agilent Technologies). A positive control (brain sections from mice experimentally infected with WNV_NSW2011_ [16]) was included in every IHC-batch performed. The sections were counterstained with Meyer’s hematoxylin and examined on a Nikon Eclipse 51 E microscope. Digital micrographs were generated as described above.

### 2.8. Serology

Quantification of anti-WNV specific antibodies was performed by two methods: blocking-ELISA [26] and microneutralization assay [16,27]. The blocking-ELISA protocol has been described extensively in previous publications [16,26,27]. The current assay used lysate from C6/36 cells infected with WNV_KUN_ strain MRM61C as the coating antigen. Monoclonal antibodies used for these competitive assays were either an anti-flavivirus envelope monoclonal antibody, 6B6C-1, or an anti-WNV NS1 specific monoclonal antibody, 3.1112G [27,28]. A cut-off of 30% inhibition was used to determine positive samples [27]. Western blot for detection of serum antibody specificities for WNV proteins was performed as described [16], but using goat anti-alligator immunoglobulin (Novus Biologicals, Centennial, Colorado) and fluorophore 680-conjugated rabbit anti-goat-immunoglobulin.

## 3. Results

### 3.1. Isolation of WNV_KUN_ from Skin Lesions and Plasma

**Figure 1:**
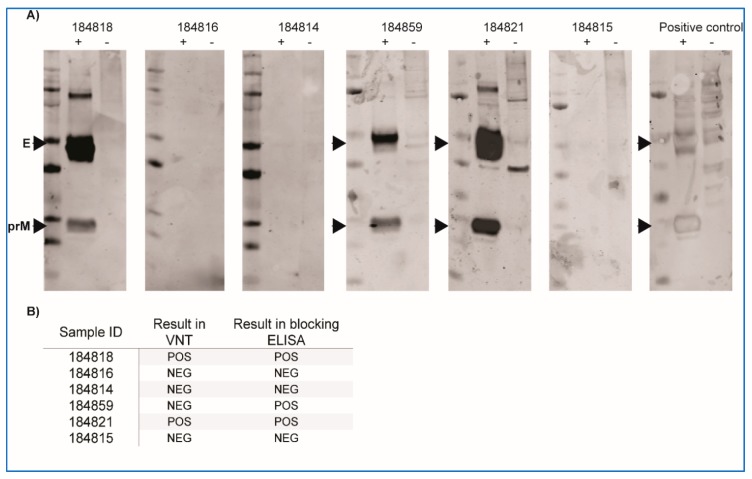
Antibody responses in farmed saltwater crocodiles with (184818, 184821) and without “pix” skin lesions. (**A**) Western blot using serum samples from crocodiles presenting with skin lesions and lesion-free animals from the same farm were used to probe WNV_KUN_ (strain MRM61C) cell lysate (+) or mock-infected cell lysate (−). Signal for envelope (E) and pre-membrane (prM) proteins are observed at approximate molecular weights of 50 and 20 kDa, respectively. WNV_KUN_-reactive horse serum was used as positive control. (**B**) Reactivity of the same serum samples in virus neutralisation test (VNT) and blocking ELISA. POS—positive, NEG—negative.

Multiple attempts at isolating WNV from skin lesions and plasma of naturally infected crocodiles and from mosquitoes caught at affected crocodile farms were made, but all were negative. Similarly, attempts at detecting viral antigen (E protein or NS1) in skin lesions of naturally infected saltwater crocodiles have so far been unsuccessful despite the proven sensitivity of this approach [16,26,29]. Nevertheless, by RNA-extraction and RT-PCR, WNV genomic material was detected in several skin lesions from naturally infected crocodiles [12]. Animals with skin lesions also had WNV-specific antibodies that reacted strongly to WNV-antigens in Western blot (Figure 1A) and in ELISA or VNT (Figure 1B).

### 3.2. Sequence Analysis of Virus RNA from Skin Lesions

**Figure 2:**
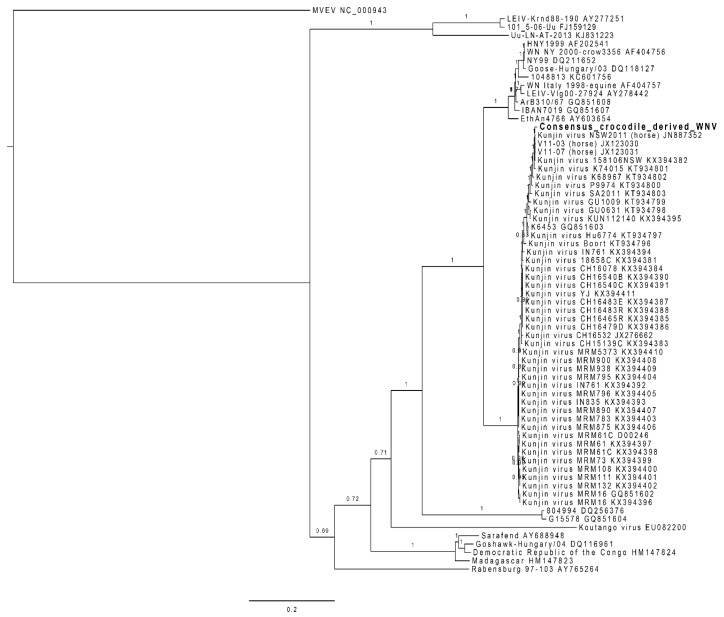
Phylogeny of the *C. porosus*-derived WNV strain (Genbank accession number: MN954648). This maximum likelihood phylogeny was built from a nucleotide alignement of the whole genome of 67 WNV sequences and one Murray Valley Encephalitis virus (NC_000943) sequence used as an outgroup, using MrBayes 3.2.6 in Geneious 8.1.9. The branch labels represent the posterior probability and the scale bar represents the substitutions per site.

The RNA from one of the skin lesions positive for WNV_KUN_ by RT-PCR was analysed by next-generation sequencing. From the data produced, only 222 reads corresponded to WNV, and did not cover the whole genome. We therefore designed primers to amplify large fragments from additional samples (D17, D63, D68, D117) by high-fidelity one-step RT-PCR, and Sanger-sequenced the missing sections. This allowed us to obtain a composite consensus sequence that covered the whole genome, despite some sequence variation between samples (Genbank accession number pending). The translated sequence obtained was included in a complete ORF comparison with the prototype WNV_KUN_ strain (MRM61C), the NSW2011 strain of WNV_KUN_ and WNV_NY99_. WNV_NSW2011_ (JN887352) was the most closely related strain to the crocodile-derived composite sequence, with a maximum 13 amino acid changes over the ORF (Table 2 and Figure 2). The amino acids for multiple WNV strains at these 13 positions are listed in Table 2 and cross-referenced with previously published data on virulence determinants for WNV_NY99_ [6,30].

### 3.3. WNV_KUN_ Replication in C. porosus Derived Cell Lines 

Since we were unsuccessful in obtaining a field isolate of the WNV circulating amongst crocodiles, an infectious clone was generated based on the crocodile-derived viral amino acid sequence described above. However, it was subsequently found that this infectious clone grew poorly or not at all in crocodile-derived cells. Furthermore, given the minor sequence differences present in the viral genomes obtained from different crocodile skin lesions compared to the NSW2011 strain of WNV_KUN_, it was decided to use this virus for all subsequent in vitro and in vivo studies, as it is a well-characterized, recent and low-passage isolate [4,6].

**Figure 3:**
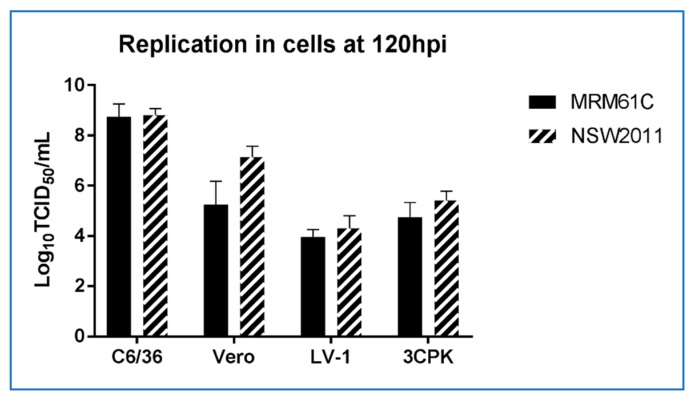
Comparison of virus replication in mosquito (C6/36), mammalian (Vero) and crocodile (1-LV, 3-CPK) cell lines.

**Figure 4:**
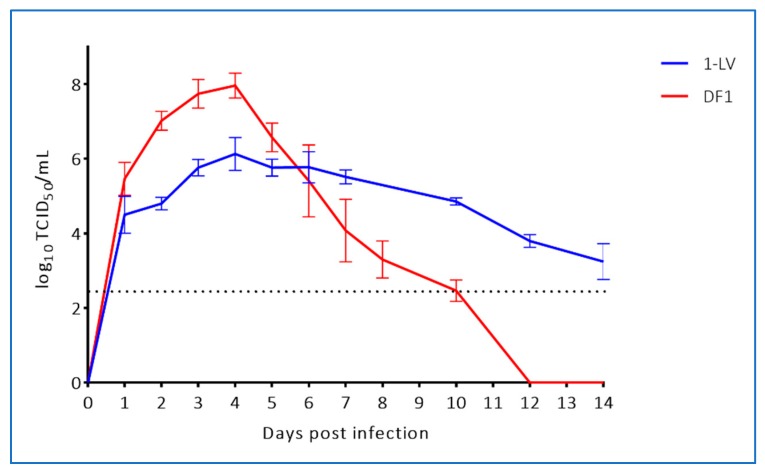
Comparison of WNV_KUN_ replication in crocodile cells (1-LV) versus avian cells (DF-1) during a two-week period. Cells were infected at a MOI = 1 and triplicate wells were terminated at the indicated time points and tested individually for viable virus by TCID_50_-assay in C6/36 mosquito cells. Stippled line indicates limit of detection.

To assess replication of WNV_KUN_ in crocodile cells we infected liver- (LV-1) and kidney- (3CPK) derived cell lines with WNV_KUN_ (NSW2011) and the prototype WNV_KUN_ strain (MRM61C) at a MOI of 0.1. For comparison, we also infected mosquito cells (C6/36) and mammalian cells (Vero) with these viruses and assessed titres at 120 hours post infection (hpi). While virus yield was lower in the crocodile cell lines compared to mosquito and mammalian cell culture (Figure 3), it should be noted that the crocodile cells replicate extremely slowly [17], indicating a potentially lower metabolic rate (G.H., W.W.S and H.B.O., unpublished data). When compared to avian cells (DF-1), the NSW2011 WNV_KUN_ strain replicated to a slightly lower titre in 1-LV cells, but due to the lesser cytopathic effect in the LV-1 cells, replication was still detectable at 14 dpi. (Figure 4). In contrast, the DF-1 cells displayed marked cytopathic effect and viral titers dropped precipitously from a peak at 4 dpi, likely due to a combination of decreasing number of viable cells and thermal inactivation of the virus (Figure 4). 

### 3.4. Experimental WNV-Infection in Hatchling C. porosus 

#### 3.4.1. Clinical Observations

**Figure 5:**
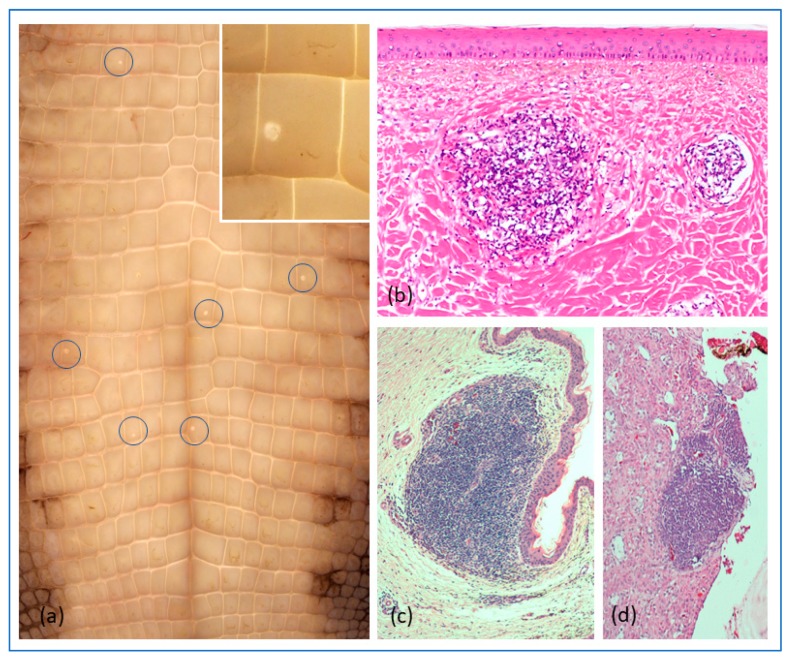
Representative gross and histopathological changes in experimentally WNV-infected *C. porosus* hatchlings. (**a**) Typical WNV-induced skin lesion (“pix”; enlarged in insert); (**b**) Microscopic appearance of a typical “pix” lesion; (**c**) Lymphofollicular aggregate in subepithelial layers of the oral cavity; (**d**) Lymphofollicular aggregate in the adrenal gland.

No adverse effects of the virus inoculation or blood samplings were observed in any of the hatchlings, and the animals appeared to grow at the expected rate. At no stage during the observation period were overt clinical signs, such as neurological disease, observed. Seven animals in the virus-infected groups developed small, WNV-like skin lesions (“pix”; Figure 5a). These were sampled at necropsy and subjected to either microscopic examination or RT-PCR for viral RNA. 

#### 3.4.2. Gross and Histopathology 

Histologically, the skin lesions appeared as noted for natural WNV-infections (Figure 5b) [12]. No frank gross lesions, apart from the skin-lesions, were observed at necropsy. Histologically, the only changes seen were development of lymphofollicular aggregates in various tissues (summarized in Table S3), notably in the subepithelial layers of the tongue and conjunctiva and in the submucosa of the gastrointestinal tract, more rarely in the kidneys and liver. In the tongue, the lymphoid aggregates were mostly surrounding small nerve bundles associated with sensory organs (taste/touch sensors), as was also the case in the skin lesions (Figure 5b,c).

#### 3.4.3. Virus Detection by qRT-PCR, Isolation and Immunohistochemistry

**Figure 6:**
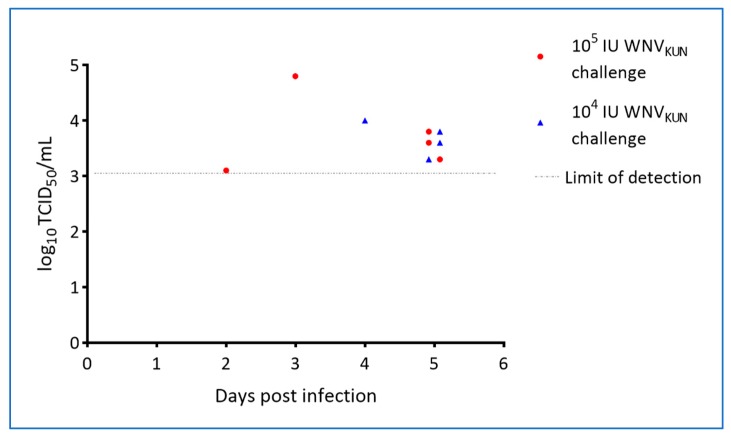
Viral titres in plasma from hatchling saltwater crocodiles following experimental WNV_KUN_ challenge. Stippled line indicates limit of detection.

**Figure 7:**
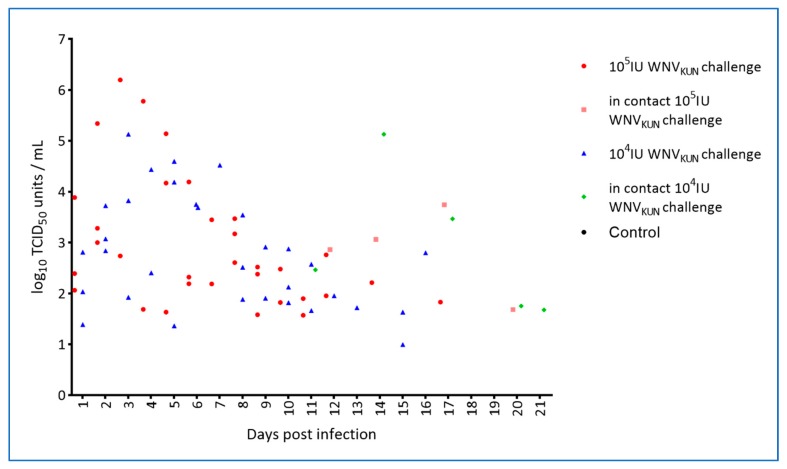
Quantification of WNV_KUN_ RNA in plasma from hatchling saltwater crocodiles following experimental WNV_KUN_ challenge or in-pen contact animals determined by qRT-PCR (limit of detection 10 TCID_50_/mL at Ct = 39; see Figure S1 and Table S4).

By inoculation of plasma samples on C6/36 mosquito cells, viremia was detected on days 2–5 in WNV-challenged animals but not in-contact animals (Table 3). For a small subset of animals, virus isolation and titrations were performed on both whole blood and on plasma, with the former possibly being slightly more sensitive as also seen for other flaviviruses [31]. However, all samples displayed some level of cytotoxicity at the lowest dilutions tested and therefore the limit of detection was relatively high (2–3 log_10_ TCID_50_/mL; Figure 6). It is thus possible that there are false negative samples in the set and, that the actual period of viremia was longer as suggested by the qRT-PCR results which showed intermittent plasma viremia in the virus challenged animals out to 17 dpi (Figure 7, Figure S2 and Table S4).

Low levels of WNV_KUN_ RNA were detected by qRT-PCR in three “pix” lesions tested by this approach. The Ct-scores ranged from 26.16 to 31.35, equivalent to approaximately 10^2^ TCID_50_/sample (refer to Table S2 for conversion).

**Figure 8:**
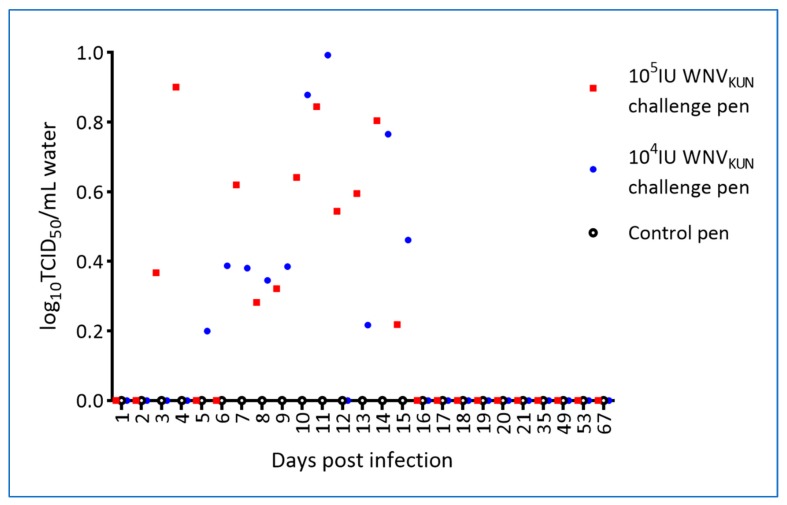
WNV_KUN_ RNA detection in pen water by qRT-PCR. Data are shown as TCID_50_-equivalents. Pen water was collected daily just prior to cleaning of the pen and RNA isolated from filters following filtration of 60 mL of water.

Water samples were collected from the pens prior to cleaning, once per month before the experimental period and daily during the experimental period, and tested by RT-PCR. Notably, WNV_KUN_ genomic material was detected in the water from the pens holding the virus-challenged hatchlings on multiple sampling days, but not in the pen holding the control hatchlings (Figure 8). 

**Figure 9:**
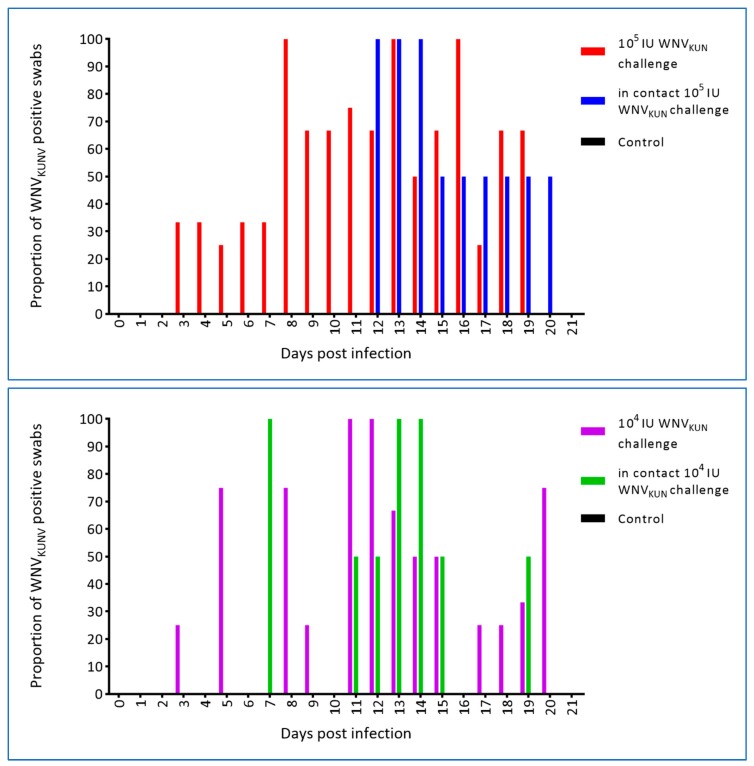
Proportion (%) of oral swabs positive for WNV RNA as determined by qRT-PCR with a cut-off of Ct ≥ 40 [19]. Two in-contact hatchlings and three or four inoculated animals were sampled and tested each day.

**Figure 10:**
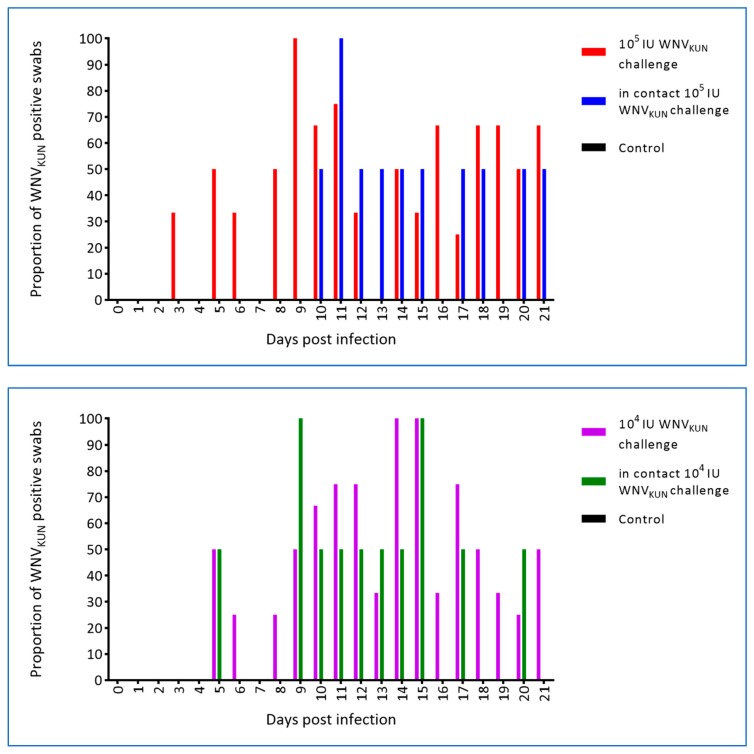
Proportion (%) of cloacal swabs positive for WNV RNA as determinaed by qRT-PCR with a cut-off of CT ≥ 40 [19]. Two in-contact hatchlings and three or four inoculated animals were sampled and tested each day.

Swabs from the oral cavity and cloaca were taken prior to infection and at each blood sampling thereafter, i.e., eight samplings per hatchling. WNV was first detected in both the oral and cloacal swabs on day 3 p.i. in the 10^5^ IU challenged crocodiles. Similarly, WNV_KUN_ was detected in the oral swabs of 10^4^ IU challenged hatchlings at 3 dpi, but was not recovered from the cloacal swabs until 5 dpi. Oral swabs of in-contact crocodiles were first WNV_KUN_-positive on days 7 and 11 p.i. for the 10^4^ IU and 10^5^ IU challenged groups, respectively, and days 5 and 10 p.i. for cloacal swabs (Figure 9 and Figure 10), although at the later time points, i.e., past day 15 p.i., the levels were low as judged by the Ct scores and no longer detectable in the pen water (Figure 8). In the 10^5^ IU group, all hatchlings had WNV-RNA-positive oral swabs on 2–6 of the eight samplings, while cloacal swabs were positive on 1–5 samplings per animal, with one animal being consistently negative. All six in-contact animals in the 10^5^ IU-pen had RNA-positive swabs on 1–3 samplings, while only four had positive cloacal swabs. In the 10^4^ IU challenged group, 10 of the animals had 1–5 virus RNA-positive oral swabs and all had RNA-positive cloacal swabs on 1–5 samplings. All in-pen contact animals in this cohort had 1–3 positive oral swabs and five had 1–4 RNA-positive cloacal swabs. Notably, positive oral and cloacal swabs were detected in both the 10^5^ and 10^4^ IU pens up to six days after the last positive tank-water-positive sample on day 15 of the experiment (Figure 8), suggesting that the viral RNA detected in the swabs was not just contamination from the pen water.

**Figure 11:**
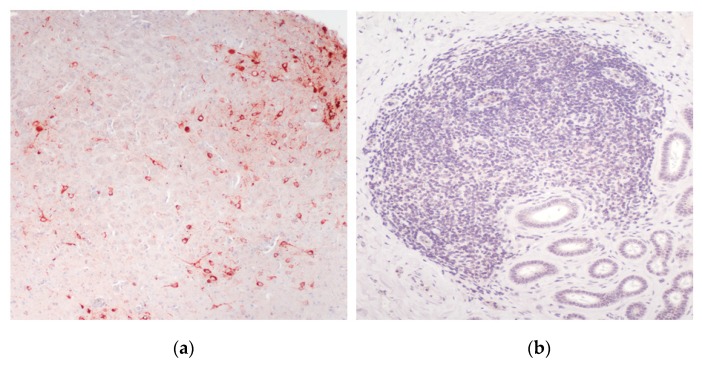
Immunohistochemical detection of WNV_KUN_ NS1, using mAb 4G4, in tissues from experimentally infected animals. (**a**) Brain from mouse experimentally challenged with the NSW2011 isolate of WNV_KUN_ [16] showing virus protein-positive neurons throughout the cerebrum (red cells). (**b**) Lymphoid aggregate in the tongue of a hatchling crocodile 21 dpi with WNV_KUN_. No virus antigen-positive cells are apparent in either the lymphoid aggregate, sublingual glands, endothelial cells or the interstitial tissue fibrocytes.

The WNV-like skin lesions (pix) that developed in three of the virus-challenged crocodiles (Figure 5) were confirmed to be WNV_KUN_-positive by RT-PCR, but no viral antigen was detected in those lesions subjected to IHC. A large number of the tissue samples with lymphoid aggregates (see Figure 5b,c,d) were also subjected to IHC for detection of WNV_KUN_ NS1, a highly sensitive assay for virus replication [16,26,29]. While the positive controls (brains from mice experimentally infected with the NSW2011 isolate of WNV_KUN_ had NS1 signal in neurons (red cells in Figure 11a), all samples from the crocodiles terminated at day 21 p.i. and at the end of the study were negative for viral protein (example in Figure 11b).

#### 3.4.4. Detection of WNV_KUN_-Specific Antibodies Pre- and Post-Infection

Pre-screening of the hatchlings for maternal antibodies (egg yolk-transmitted) specific for WNV_KUN_ was conducted by virus-neutralization test (VNT) at BVL, and only animals negative in the VNT at 2–3 months of age were used for virus challenge. Two crocodiles with very low or query WNV_KUN_ antibody titers were assigned to the non-infected control group. These crocodiles showed persistent low WNV_KUN_ antibody titers for the entire 27 weeks of observation and were from the same clutch of eggs.

**Figure 12:**
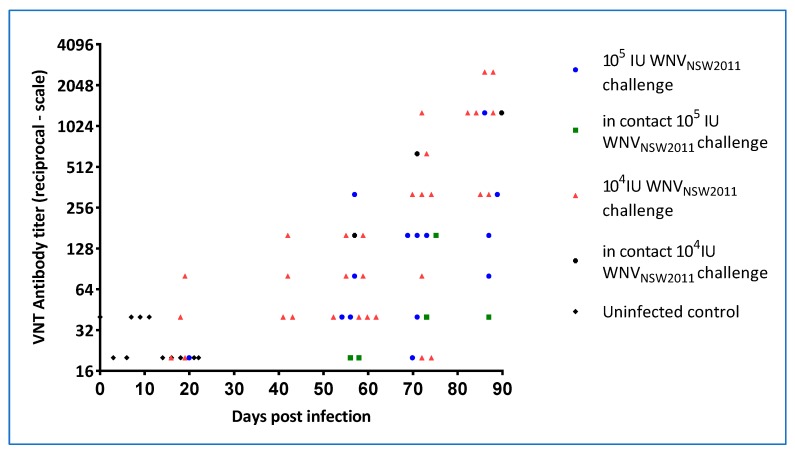
WNV_KUN_-neutralizing antibody responses in experimentally infected hatchling saltwater crocodiles and in the in-pen contact animals. For the first 21 dpi, each animal was bled every third day. At the later time points (after 40 dpi), all remaining animals were bled on each occasion.

Post-challenge antibody levels were assessed by a WNV_KUN_-specific and flavivirus group reactive monoclonal blocking-ELISA (data not shown) and by VNT. All animals in the 10^4^ IU group developed WNV_KUN_-neutralizing antibodies (Figure 12), with most having detectable levels by 21 dpi. Similarly, all but one animal in the 10^5^ IU group developed neutralizing antibodies, albeit for most delayed relative to the 10^4^ IU group (Figure 12). In both groups, the titers continued to rise beyond the time of detectable viremia and cloacal virus shedding, suggestive of a persistent source of viral antigen. Notably, three in-contact animals (one in the 10^4^ IU and two in the 10^5^ IU group, respectively) seroconverted—first detectable eight weeks after the experimental infections but then with continuing rising titers throughout the remainder of the experimental period (Figure 12). 

## 4. Discussion

We have presented data that confirm that WNV does indeed cause characteristic skin lesions, so-called “pix”, in a subset of infected saltwater crocodiles within a few weeks of infection and that at least some of the infected animals sustain a viremia at a level sufficient for transmission to biting mosquitoes [32]. Moreover, we have demonstrated that the virus is shed into the water, likely via fecal material, where it can spread to in-contact animals, presumably via mucosal infection—either fecal-oral or via other exposed mucosal surfaces such as conjunctiva or the nasal cavity. Regardless of viral challenge dose, WNV-neutralizing antibodies appeared in the directly challenged animals around 21 dpi, at which time viremia had ceased as determined by qRT-PCR, but cloacal shedding may still have taken place (Figure S2). The in-pen controls, that became infected, developed WNV-neutralizing antibodies detectable around six weeks into the experiment. Viremia was detected in the in-contact animals by RT-PCR on plasma samples between day 11 and 21, suggesting that these animals received a much lower infection dose (via the mucosal route) than that of the inocula resulting in a different transmission-clearance dynamic and possibly different or additional antiviral mechanisms involved [33,34,35]. The time of viremia in the in-contact animals does appear to correlate with detection of virus genomic material in oral and cloacal swabs of the virus-challenged hatchlings as well as in the pen-water (Figure 9, Figure 10 and Figure S2), but since tank-water was changed daily, there was no actual build-up of infectious material. The in-contact animals would nevertheless have been repeatedly exposed during that period and potentially re-infected until activated innate responses [36] or virus-specific antibodies [35] interfered with this transmission route. The noted discrepancy between viremia, as detected by virus isolation versus qRT-PCR (Table 3, Figure 6 and Figure 7), suggests that past 5 dpi virus in the plasma may have been in antibody–virus complexes [37] or toxicity of the crocodile plasma may have resulted in false negative isolation results. This issue will be addressed in future studies.

While WNV has been associated with severe disease outbreaks in both wild and farmed American alligators in several USA states (reviewed in [8,10]), there are no reports of similar disease outbreaks amongst crocodilians in other parts of the world, including Northern Australia and South-East Asia, where *C. porosus* also occur. American alligators display clinical neurological signs and suffer severe gastrointestinal lesions, including stomatitis and necrohemorrhagic enteritis following infection with WNV_NY99_ [10]. High viral titers were detected in the liver of the alligators [32], suggesting that some of the virus detected in the cloacal swabs and water from this study might have originated from this organ via bile secreted into the gastrointestinal tract. While we observed development of lymphofollicular proliferations in various mucosal tissues as well as liver and kidney of the WNV_KUN_ infected saltwater crocodiles and virus RNA was detected in cloacal swabs and tank-water, no frank pathology was observed in any of the tissues and organs examined. Furthermore, no viral antigen was detected in association with these lymphoid aggregates using IHC. Thus, whether the presence of these lymphoid proliferations reflects prior replication of virus in the sites or simply is a reflection of a stimulated immune system in the infected animals cannot be established based on the present study. Future studies should include daily tissue sampling of virus-challenged animals during at least the first 15 dpi, i.e., a kinetic study, and employ additional methodologies such as in situ hybridization, RT-PCR and virus isolation from tissues.

The one commonality between WNV_NY99_ infection in American alligators and WNV_KUN_ infection of saltwater crocodiles, is the development of typical skin lesions, “pix”, consisting of lymphohistiocytic proliferations in the superficial dermis, with attenuation of the overlaying epidermal layers and degeneration of the dermal collagen (Figure 5) [10,11,12]. Previous studies in alligators have failed to detect virus in skin lesions, and while we have previously successfully detected WNV_KUN_ viral RNA in “pix” lesions from naturally infected saltwater crocodiles [12] and did so from a few lesions in the experimental animals in this study, our attempts at isolating replication competent virus in cell culture has so far been fruitless. Nor have we been able to detect viral nonstructural (NS1) or envelope (E) protein expression by IHC in either these skin lesions or other tissues. However, the presence of viral RNA in some of these lesions suggests that either the virus does indeed replicate in the site, albeit likely only very early in the infection, or inactivated viral particles in dermal dendritic cells and macrophages stimulate an in situ immune reaction, which persists long enough to cause permanent damage to the dermis and resulting in grossly apparent “pix” lesions. Again, a kinetic study of the virus infection with extensive sampling of animals may allow elucidation of this aspect.

Knowledge about the antiviral immune defenses in reptilians in general and crocodilians in particular is still relatively limited [36], and the role of co-infections has so far not been systematically explored. While the hatchlings used in this experiment were kept under strict quarantine conditions, crocodiles in the wild and on farms are exposed to a plethora of other infectious agents, including viral, fungal, bacterial and parasitic (reviewed in [7,8,38,39]), some of which, notably herpes-, rana- and retroviruses, are known to affect the immune system profoundly [40,41,42]. While WNV_KUN_ is less virulent than WNV_NY99_, at least in horses, mice and birds [6,16,26], it may not be the only explanation for the dramatic difference in clinical outcomes of WNV infection in alligators and saltwater crocodiles, respectively. The Kunjin strains of WNV have presumably circulated in Australia for a very long time [4], and Australasia is host to an abundance of related flaviviruses, such as Murray Valley encephalitis virus and Alfuy virus, that may confer some degree of cross-protection [5,43,44]. Hence, *C. porosus* may have evolved innate immune defenses to this group of viruses, which ensures relatively fast elimination of the virus without a severe inflammatory response. In contrast, WNV_NY99_ is a relatively newly introduced infectious agent in the Americas and native crocodilians may not have been through a similar evolutionary pathway for this group of viruses. Notably, antibodies to St. Louis encephalitis virus, a flavivirus circulating in southern USA and South America, have never been detected in crocodilians [7,8,45]. Future studies of the host-virus interaction in WNV-infection of crocodilians should therefore also focus on innate and adaptive antiviral defenses as well as the potential role of co-infections and the gut microbiota [8,46,47]. In this study, we detected maternal antibodies to WNV in a number of the clutches used, i.e., passive immunity acquired through the egg yolk, which persisted beyond 4 months in a few animals. Other seeming clutch-effects noted related to the kinetics of the infection-elicited antibody response and the development of “pix” lesions; however, a study with larger group sizes will be required to determine whether a true immunogenetic influence also play a role in the pathogenesis of the lesions.

## 5. Conclusions

While WNV_KUN_ infection in saltwater crocodiles appears to be a relatively innocuous event, it does have serious economic ramifications for tropical regions of Australia and South-East Asia, where farming of *C. porosus* for the production of hides is an important and growing industry, benefitting local communities and, interestingly enough, encouraging conservation efforts for this iconic apex predator. It is therefore imperative that ways are found to prevent WNV-infection, at least in the farm setting. While mosquito and bird management measures, notably the latter, may go some way to address this, the fact that the virus is easily and efficiently transmitted horizontally between pen mates via the water, suggests that vaccination is necessary to truly prevent transmission, whether by mosquitoes or via water contact. In addition, if genetics indeed plays a role, then methodologies to assess overall vulnerability of ranched stock to WNV “pix” lesions should be pursued. 

## Figures and Tables

**Table 1:** West Nile Virus, Kunjin strain (WNV_KUN_) experimental infection trial groups description and size.

Group	Control (No Injection)	1 × 10^5^ IU ^1^ WNV_KUN_ Challenge	In-Contact Controls for 10^5^ IU Injected	1 × 10^4^ IU WNV_KUN_ Challenge	In-Contact Controls for 10^4^ IU Injected
N	7	10	6	11	6

^1^ IU = infectious units.

**Table 2:** Comparison of amino acid sequences between the crocodile-derived WNV RNA and three previously described WNV strains over the 13 amino acid positions that differed between WNV_KUN_ (strain NSW2011) and the crocodile-derived WNV sequence.

Position in Polyprotein ^1^	Protein	Position in Protein	WNV_ny99_	WNV_kun_ (mrm61c)	WNV_kun_ (nsw2011)	Crocodile-Derived WNV	nt Position in Assembly
**20**	C	20	G	G	G	**E**	104
**108**	prM	3	K	K	R	K	404
**166**	prM	61	Y	H	Y	H	**578**
**207**	prM	102	T	T	T	**A**	**701**
**837**	NS1	46	I	I	V	I	2591
**1026**	NS1	235	G	G	G	**E**	3158
**1192**	NS2A	49	I	I	I	V	**3656**
**1355**	NS2A	212	L	F	F	**L**	4145
**1719**	NS3	214	N	N	N	S	5237
**2210**	NS4A	86	V	V	V	I	**6710**
**2683**	NS5	155	E	E	E	Q	8129
**2797**	NS5	269	K	K	K	R	8471
**2978**	NS5	450	H	H	H	Y	9014

^1^ Amino acid position numbers highlighted in red-brown are differences from WNV_NSW2011_ which are predicted to have an influence on protein structure or function.

**Table 3:** Detection of viremia in WNV-challenged and in-contact hatchling crocodiles by inoculation of C6/36 cells with plasma (positive/tested).

Group	Day p.i.									
	0	1	2	3	4	5	6	7	8	9
10^5^ IU	0/10	0/3	1/4	1/3	1/3	2/4	0/3	0/3	0/2	0/3
10^5^ IU in-contact	0/6	0/2	0/2	0/2	0/2	0/2	0/2	0/1	0/2	0/2
10^4^ IU	0/11	0/3	0/4	0/4	0/3	3/4	0/4	0/3	0/2	0/4
10^4^ IU in-contact	0/6	0/2	0/2	0/2	0/2	0/2	0/2	0/2	0/1	0/2
Control	0/7	0/2	0/2	0/2	0/2	0/2	0/2	0/2	0/1	0/2

## Supplementary Materials

The following are available online at https://www.mdpi.com/1999-4915/12/2/198/s1, Table S1: Primers used for the “fill in” to attain the full-length genome sequence of the *C. porosus* derived WNV strain. Table S2. Standard for estimation of TCID_50_-equivalents of WNV_KUN_ in pen-water as determined by qRT-PCR. Table S3: Summary of histopathological changes in tissues and organs from experimentally WNV-infected hatchling saltwater crocodiles. Table S4. Plasma viremia as detected by qRT-PCR and converted to TCID_50_ equivalents. Figure S1. Standard curve to determine WNV_KUN_ infectious unit equivalents from plasma qRT-PCR CT scores. Figure S2. Relationship between virus detection and antibody development.
