# Supplementary material for: Mosquito-Independent Transmission of West Nile virus in Farmed Saltwater Crocodiles (Crocodylus porosus)
Source: Viruses. 2020 Feb 11;12(2):198. doi: 10.3390/v12020198 (PMC7077242; doi:10.3390/v12020198)
Supplement: Supplementary file 1 [file viruses-12-00198-s001.pdf]

Table S1. Primers used to complete the sequencing of the crocodile-derived WNV.

CROCKUNV3F	GATGCGGAAATCACAGGTTCC
CROCKUNV3R	CAGCCAGCCCATCTCATTGG
CROCKUNV4F	CTGAAATCATTCAAAGACTTCGC
CROCKUNV4R	GCCTCACGCTCTTCATCCACC
CROCKUNV5F	GGAACGAGAGCAGTGGGAAGAC
CROCKUNV5R	TGCGGCACGGGGTCTCCACTAACC
CROCKUNVseq1F	CCTGATTGCTGGTGTGG
CROCKUNVseq1R	GCTGTCACCTTCAAGGACC
CROCKUNVseq2R	GGAATGGCCATAGAGTCC
CROCKUNVseq3F	GGAACAGTTTGGAGGTGG
CROCKUNVseq3R	CCAAGAACACGACCAGAAGG
CROCKUNVseq4F	GCTGATGTCTCCTCATAGG
CROCKUNVseq4R	CCCAGTCATCGTTCTTGC
CROCKUNVseq5F	CGTACCACACCCATCATGC
CROCKUNVseq5R	GGTGTCTGAATTGAGTAGAGG
CROCKUNVseq6R	CCAGAGTATGGAACATCGC
CROCKUNVseqEF	GGAGTTTGAAGAACCACATGC
CROCKUNVseqER	CCCTGAGTTATCCAAGACATG
WNV NS1 F	CATGCTGACACTGGATGTGCCATAG
WNV NS2B R	CTCCTCTCTTTGTGTATTGGAGAGTTATC

Table S2. Standard for estimation of $TCID_{50}$-equivalents of WNV_{KUN} in pen-water as determined by qRT-PCR.

Standard	Log 10 (standard)	CT score
$10^{5.92}$ $TCID_{50}$/ml (neat)	5.92	12.788361
1:10	4.92	18.738192
1:100	3.92	22.422401
1:1000	2.92	25.790836
1:10000	1.92	28.877739
1:100000	0.92	32.220005
1:1000000	-0.08	36.105015
1:10000000	-1.08	39.028511

Table S3. Summary of histopathological changes

Tissue/ Organ	Tongue	Conjunctiva	Kidney	Stomach	Intestine	Liver	Lung	Brain	Pancreas	Adrenal gland
Group										
Control		Transepithelial lymphoid aggregate in the lachrymal glands	Rare minute interstitial lymphoid infiltrates				Lymphoid aggregates in lung interstitia			
10^5 IU	Locally extensive lymphoplasmacytic infiltrate with high endothelial postcapillary venules<br><br>Lymphoplasmacytic infiltrates with activated dendritic cells & transendothelial migrating cells	Lymphoplasmacytic perivascular, non-glandular consistent with focal reaction to insult	Interstitial lymphoplasmacytic & histiocytic infiltrate in the interstitium consistent with interstitial nephritis<br><br>Focal lymphoplasmacytic in the renal medulla	Activated lymphoplasmacytic infiltrates in the mucosa and submucosa		Multifocal lymphoplasmacytic infiltrate in the portal area<br><br>Blood clot in the portal area with no signs of inflammation suggesting an iatrogenic incident. There is an infiltrate of dead and dying cells in the affected areas	Congestion with a mononuclear cell infiltrate	Gliosis		

10^5 IU in-contact control	Locally extensive lymphoplasmacytic infiltrate with high endothelial postcapillary venules	Lymphoplasmacytic aggregate with transepithelial migration and damage on the epithelium	Focal lymphoplasmacytic in the renal medulla	Multifocal lymphoplasmacytic & histiocytic infiltrates with dominance of lymphocytes (lymphoid aggregates) with a few monocytes in the mucosa of the stomach	Mucosal lymphoplasmacytic infiltrate	Lymphoplasmacytic heterophilic infiltrate	Interstitial lymphoplasmacytic infiltrate	Lymphoplasmacytic & heterophilic infiltrate with activated dendritic cells		Lymphoplasmacytic infiltrate
10^4 IU	Lymphoplasmacytic histiocytic infiltrate with high endothelial post capillary venules with a mixed population (migrating) cells<br><br>Vascular centric lymphoplasmacytic infiltrate	Subepithelial lymphoplasmacytic histiocytic infiltrate with activated high endothelial post capillary venules<br><br>Lymphoplasmacytic infiltrate compressing the epithelium		Subepithelial multifocal lymphoplasmacytic histiocytic infiltrate compressing the crypts with activated high endothelial post capillary venules		Multifocal lymphoplasmacytic infiltrate in the portal area	Vascular centric and interstitial lymphoplasmacytic infiltrate	Lymphoplasmacytic histiocytic infiltrate	Lymphohistiocytic infiltrate with isolated dying cells	

	Histiocytic infiltrate destroying the lachrymal gland	in the lachrymal gland								
10^4 IU in-contact control	Focal lymphoplasmacytic infiltrate in the mucosa-muscularis plagued endothelial cells, transepithelial and surrounding vessels		Lymphoplasmacytic infiltrate along with exudative accumulation in the vascular lumen			Locally extensive lymphohistiocytic infiltrate			Activated lymphoplasmacytic infiltrated compressing the pancreatic acini	

Table S4. Plasma viremia as detected by qRT-PCR and converted to $TCID_{50}$ equivalents.

		D1	D2	D3	D4	D5	D6	D7	D8	D9	D10	D11	D12	D13	D14	D15	D16	D17	D18	D19	D20	D21
Challenge	Animal ID	TCID50/ml	TCID50/ml	TCID50/ml	TCID50/ml	TCID50/ml	TCID50/ml	TCID50/ml	TCID50/ml	TCID50/ml	TCID50/ml	TCID50/ml	TCID50/ml	TCID50/ml	TCID50/ml	TCID50/ml	TCID50/ml	TCID50/ml	TCID50/ml	TCID50/ml	TCID50/ml	TCID50/ml
10^4	11	641.65933			27331.0681			33116.8209			65.4971252			52.4808256								
10^4	39	108.224226			253.031085						749.306707											
10^4	55	24.3462745									133.08567						627.599163					
10^4	8		5304.1636			15369.1579			76.4851891													
10^4	9		690.841429			22.9495105			325.255764			374.253039										
10^4	14		1175.73695			39248.0892			3481.73657			45.6100265										
10^4	6			134726.175			5626.98813									9.81576236						
10^4	22			83.572997						80.5432308			89.9787254			42.6769968						
10^4	23			6608.77212			4890.30183			812.719848												
10^4	57																					
10^4 ctr	45																					
10^4 ctr	67																					
10^4 ctr	33											291.148268			133734.85			2916.20899			56.5033918	
10^4 ctr	37																					
10^4 ctr	27																					
10^4 ctr	54																					47.3257227
10^5	12	245.665592			48.7446323			153.134001			299.877413											
10^5	30	115.66207									65.9826304											
10^5	32	7673.37126			598885.714			2810.48787														
10^5	13		999.419167			14697.2533			2937.82573			78.7783515										
10^5	17														162.454115			67.4608459				
10^5	35		219350.918			137744.459			1478.2202			37.0897167										
10^5	62		1900.16037			42.6769968			402.938899													
10^5	15						154.269125			330.085629			574.372613									
10^5	29			545.433768			208.824668			238.514502			89.9787234									
10^5	38			1575937.82			15477.0392			38.2017326												
10^5 ctr	3																					
10^5 ctr	60																					
10^5 ctr	20														1149.97306			5503.68844			48.029938	
10^5 ctr	36																					
10^5 ctr	1																					
10^5 ctr	40												727.495106									
control	24																					
control	46																					
control	19																					
control	53																					
control	41																					
control	47																					
NI	51																					

Figure S1.

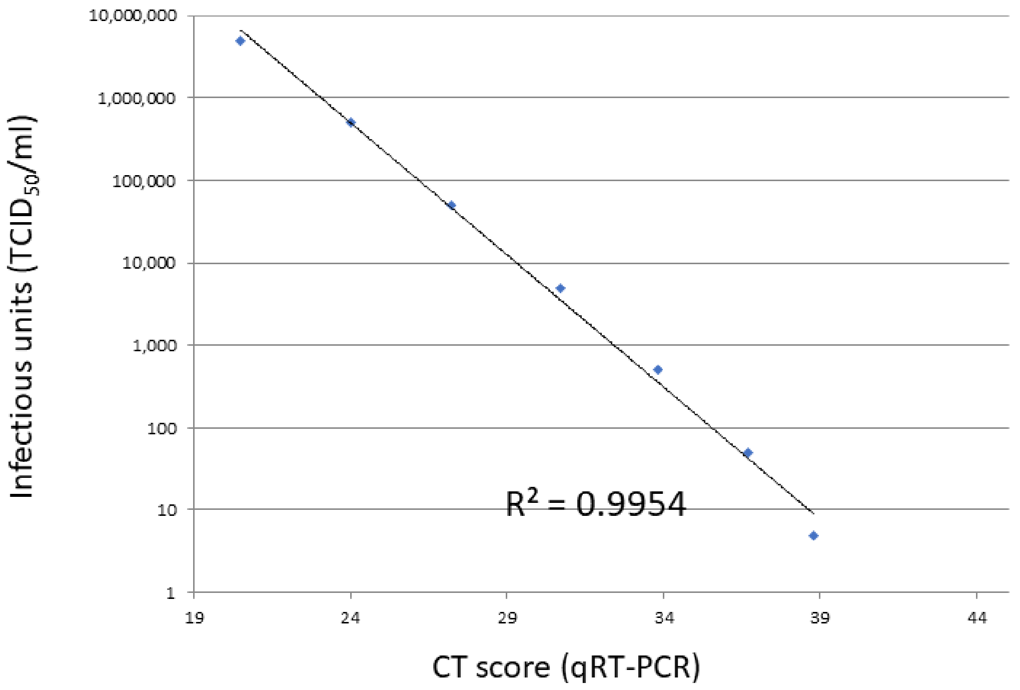

Figure S1. Standard curve to determine WNV_{KUN} infectious units equivalents from plasma qRT-PCR CT scores. Ten-fold dilutions of WNV_{KUN} (10^{-1} to 10^{-7}) were simultaneously assessed for infectious titre by $TCID_{50}$ assay and levels of viral RNA by Taqman qRT-PCR. An exponential trend line was generated from the derived CT scores and calculated infectious units of the standard dilution series using the Excel Growth Function. Infectious unit equivalents were then predicted for each plasma sample from their derived CT scores. R^2 value indicates line of best fit (closest to 1).

Figure S2.

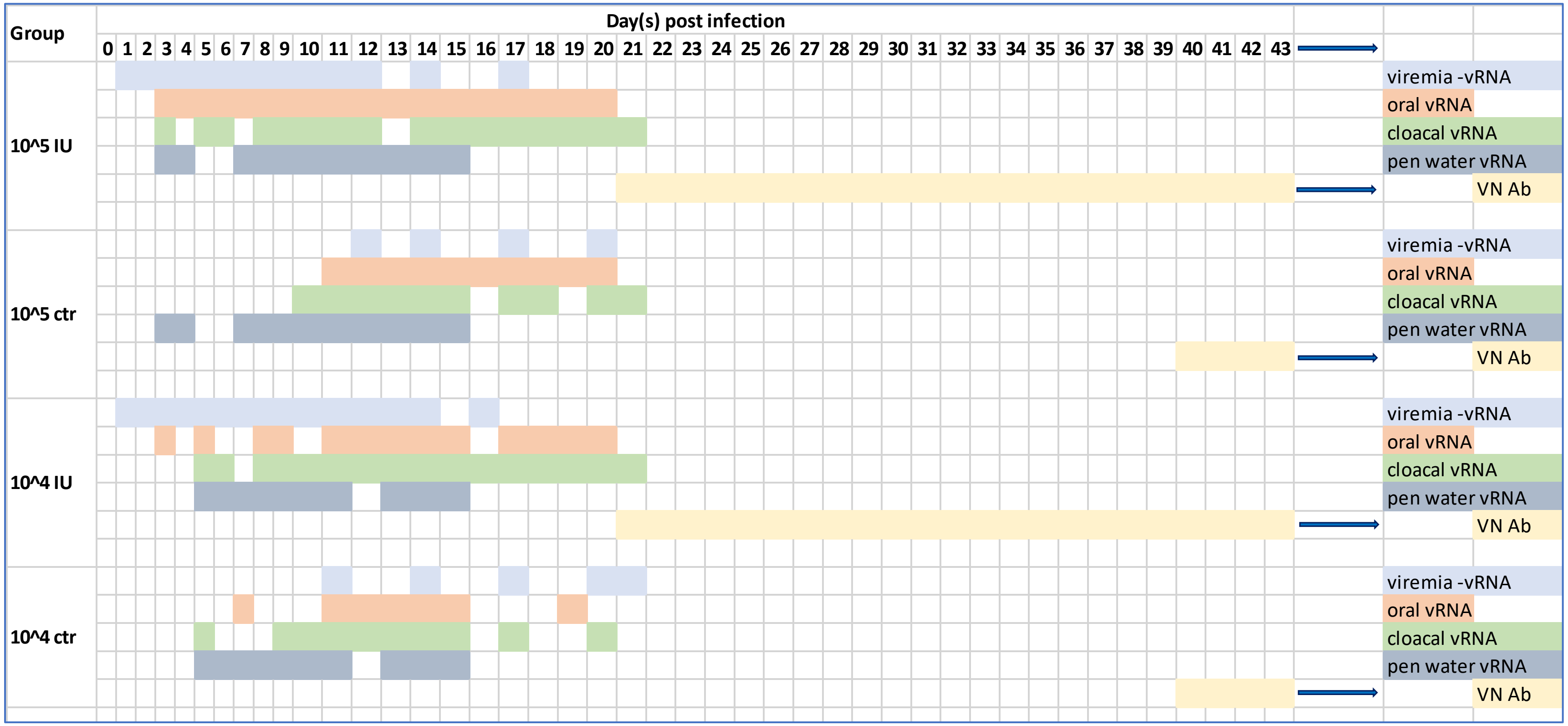

Figure S2. Overview of relationship between first detection of virus-neutralizing antibodies (VN Ab) and viremia as detected by RT-PCR, viral RNA (vRNA) in pen-water and oral and cloacal swabs. The notable delay in VN Ab development in the in-contact animals is suggestive of a different virus-host dynamic in these animals compared to hatchlings inoculated directly with WNV_{KUN}.
